# Trans-sodium crocetinate ameliorates Parkinson-like disease caused by bisphenol A through inhibition of apoptosis and reduction of α-synuclein in rats

**DOI:** 10.22038/ijbms.2024.81157.17567

**Published:** 2025

**Authors:** Majid Keshavarzi, Bibi Marjan Razavi, Hossein Hosseinzadeh

**Affiliations:** 1 Department of Pharmacodynamics and Toxicology, Faculty of Pharmacy, Mashhad University of Medical Sciences, Mashhad, Iran; 2 Targeted Drug Delivery Research Center, Pharmaceutical Technology Institute, Mashhad University of Medical Sciences, Mashhad, Iran; 3 Pharmaceutical Research Center, Pharmaceutical Technology Institute, Mashhad University of Medical Sciences, Mashhad, Iran

**Keywords:** Alpha-synuclein, Apoptosis, Autophagy, Bisphenol A, Parkinson’s disease, Saffron

## Abstract

**Objective(s):**

Trans-sodium crocetinate (TSC) is one of the crocetin derivations that is more soluble and stable than crocetin and its cis form. It easily crosses the blood-brain barrier. TSC has neuroprotective effects. Bisphenol A (BPA) is an endocrine-mimicking compound that induces Parkinson-like disease by impacting the dopaminergic system. In this research, the effects of TSCs on BPA-induced Parkinson-like symptoms via behavioral and molecular assays have been investigated.

**Materials and Methods:**

Male Wistar rats received BPA (75 mg/kg, gavage), TSC (10, 20, and 40 mg/kg), and levodopa (L-dopa) (10 mg/kg) via intraperitoneal injection (IP) for 28 days. Parkinsonian-like motor features were evaluated using bar test, rotarod, and open field experiments. Malondialdehyde (MDA) and glutathione (GSH) levels were also measured as the most important indicators of oxidative stress. Western blotting was performed for the molecular assays of alpha-synuclein (α-syn), Bcl-2, Bax, caspase-3, Beclin, and LC3 I/II proteins.

**Results:**

Our analyses indicated that treatment with TSC at high dose reduces MDA levels and protects GSH reserves. TSC can also increase anti-apoptotic Bcl-2 and decrease pro-apoptotic Bax and caspase-3 proteins. While it does not affect autophagy markers, TSC decreased α-syn protein expression, reduced the catalepsy time, and improved the time spent staying on the rotating bar and the locomotor activity.

**Conclusion:**

Overall, TSC likely ameliorates BPA-mediated Parkinson’ s-like symptoms by suppressing oxidative stress inhibition. This leads to reduced α-syn expression, which ultimately results in apoptosis inductions. Therefore, TSC can serve as a promising exploratory target for future research aimed at controlling Parkinson’s disease.

## Introduction

Saffron (*Crocus sativus* L.) is a herbaceous plant cultivated in various countries, including Iran, India, and Greece (1). The most important compounds of saffron are crocin, crocetin, safranal, and picrocrocin. Studies have reported that crocin effectively improves Parkinson-like symptoms in rats by reducing apoptosis and α-synuclein expression (2, 3). Crocetin (8,8′-diapocarotene-8,8′-dioic acid) is a low-molecular-weight carotenoid with diverse therapeutic effects (4) such as anticancer (4), antihyperlipidemia (5), antiatherosclerosis (6), and anti-inflammatory (7). The underlying mechanisms of therapeutic effects of crocetin include antioxidant properties, increasing oxygenation in hypoxic tissues, suppression of pro-inflammatory mediators, inhibition of proliferation, and induction of apoptosis in cancerous cells (8). It has been reported that crocetin protects neurons and prevents Parkinson’s disease through its antioxidant properties (9, 10). In addition, crocetin suppressed the destruction of dopaminergic neurons caused by 6-hydroxydopamine and prevented the decrease of dopamine levels (10).

Trans sodium crocetinate (TSC), as one of crocetin derivatives, reduces plasma resistance by changing the intermolecular forces of water molecules. It also increases oxygen diffusion through the plasma, enhancing oxygen supply to the tissues. TSC differs from other carotenoids due to polar groups at both ends of its hydrophobic carbon chain (11). TSC has neuroprotective effects. It has been reported that crocetin clears amyloid-beta by inducing autophagy through the AMPK pathway, and TSC can increase the degradation of amyloid-beta in monocytes of Alzheimer’s patients (12). 

Among common neurodegenerative disorders, Parkinson’s disease (PD) holds the second rank worldwide, following Alzheimer’s disease. It affects around ten million people globally. The destruction of dopaminergic neurons in the substantia nigra region of the brain is identified as the primary cause of this disease. These neurons release dopamine, a neurotransmitter that regulates motor function by transmitting messages from the substantia nigra to the motor areas of the brain (13, 14). Destruction of 60–80% of dopamine-secreting cells leads to the onset of Parkinson’s motor symptoms (15). It has been reported that factors such as oxidative stress, inflammatory factors, aquaporin 4, α-synuclein (α-Syn) accumulation, and apoptotic pathways play roles in the pathogenesis of Parkinson’s disease(16, 17). Autophagy has also been linked to Parkinson’s disease through α-synuclein (2). α-Syn is a soluble protein that accumulates in Lewy bodies as insoluble masses (18). The overexpression of α-Syn, a hallmark observed in both familial and sporadic cases of PD, as well as in several severe neurodegenerative diseases, inhibits autophagy at the early stages of autophagosome formation (19). Additionally, α-Syn can induce progressive degeneration of dopaminergic neurons and apoptosis in rodents (20). 

PD is a progressive chronic disease that ultimately leads to death. Therefore, finding a therapeutic approach to control or treat this disease is paramount. Various clinical strategies such as pharmacotherapy, surgery, deep brain stimulation, and gene therapy have been described for the treatment of PD. Among them, pharmacotherapy, especially L-dopa, is the most common (2, 21). However, L-dopa has been documented to have several side effects (21). Therefore, exploring the potential of natural products, with their therapeutic effects or supporting roles for existing medicines, is considered for treating PD (22).

Bisphenol A (BPA) is among several endocrine-disrupting compounds that mimic the action of hormones (23, 24). Additionally, BPA is a fundamental component in the production of plastics, including polycarbonate and epoxy resins (25). This compound exerts toxicity by various mechanisms such as oxidative stress leading to DNA and cell membrane damage, the inhibition of nerve transmission resulting in the inhibition of proliferation and differentiation of neural stem cells, and the induction of cell death (26-28). Conditions such as increased heat and acidity can trigger hydrolysis of the ester bond between bisphenol monomers, ultimately causing its release from containers and equipment into food and the environment (29). BPA induces apoptosis by activating caspase 3, increasing the expression of the pro-apoptotic Bax gene, and triggering the c-Jun N-terminal kinases (JNKs) pathway due to elevated levels of free radicals (30, 31). Furthermore, this compound can disrupt synaptic function, significantly affecting memory processes (32, 33). Chronic exposure to BPA is directly associated with changes in the central nervous system, especially the dopaminergic system (13, 34). 

Considering the positive effects of saffron and its compounds on the dopaminergic system, along with the favorable physicochemical properties and neuroprotective effects of TSC, the present study was conducted to investigate the impact of TSC on Parkinson-like disease induced by BPA in rats.

## Materials and Methods

***Chemicals***

BPA (Ca NO. 803546), thiobarbituric acid (TBA) (Ca NO. 108180), glycine (Ca NO. 104201), tris (hydroxymethyl) aminomethane (CaN. 108387), tris hydrochloride (Tris HCL) (Ca NO. 648317), and acrylamide (Ca NO. 800830) were purchased from Merck, Germany. The TSC (Ca NO. t59123009908) was obtained from Tinab Chem Middle East Company, Iran. The 5,5′-dithiobis-(2-nitrobenzoic acid) (DTNB) (Ca NO. D218200) was purchased from Sigma-Aldrich, and other compounds were supplied from Merck, Germany. 

***Animal***

In this study, we used male Wistar rats at 10–12 weeks of age purchased from the Center of Reproduction and Maintenance of Laboratory Animals (Mashhad University of Medical Sciences, Mashhad, Iran). Rats were grouped (a set of 7 rats per cage) and, one week before experiment initiation, acclimated to the new home cage under temperature (22–24 °C) and humidity (40–60%) conditions with a dark/light cycle of 12 hr. Animal maintenance conditions and all the steps for conducting experiments were met following the ethics committee guidelines of the Mashhad University of Medical Sciences (ethics code: IR.MUMS.AEC.1401.046).

Compounds including Olive oil (Vehicle) and BPA (75 mg/kg bw) were gavaged orally, and distilled water, TSC (10, 20, and 40 mg/kg bw) were injected intraperitoneal (35). Based on previous studies, L-dopa (10 mg/kg bw) was also injected intraperitoneal (36). BPA dose was extrapolated using a pilot test (at the 75 mg/kg dose, all rats showed Parkinsonian-like symptoms) (37). All compounds were prepared fresh daily and used for 28 days. Behavioral tests were performed 24 hours after receiving the last dose of compounds. After completing the behavioral tests, the animals were anesthetized using ketamine and xylazine (70/10 mg/kg bw) (38), the brain was removed, and the striatum region was isolated. A part of the isolated tissue was used to measure lipid peroxidation and GSH content, and another part was saved at -80 °C for western blot analysis.

***Behavioral experiments***

**Catalepsy test**

On the 29^th^ day of the experiment, a catalepsy test was performed to evaluate muscle rigidity caused by PD. The apparatus used in this test was a wooden box with a stainless bar in the middle. The longer the time to hold the bar, the more severe the catalepsy (39). 

**Rotarod performance test**

The Rotarod test is a common test to assess coordination and movement balance. The speed gradient of the rod used in the rotarod apparatus is adjusted from 5 to 30 rpm. This test includes two parts: 1) the training stage and 2) The test phase. The average animal movement on the rotating rod was estimated as a criterion for maintaining balance and muscle stiffness (40, 41).

**Open field test**

The open field test was conducted using a Plexiglas open field, including a square with 100 x 100 cm2 dimensions and walls 50 cm in height. Then, factors such as the number of passes through the central zone (50 x 50 cm), the peripheral areas, and the total number of passes were measured. The extent of stopping and the distance traveled show the severity of muscle contraction and stiffness (38).

***Biochemical experiments***

**Lipid peroxidation**

This method is based on the reaction of malondialdehyde (MDA), which is a lipid peroxidation product, with thiobarbituric acid (TBA) and the formation of a colored complex. To perform this method, phosphoric acid and TBA (0.6%) were added to the 10% homogenized samples. Then, the complex was placed in a boiling water bath for 45–90 min. After cooling at room temperature, n-butanol was added, and finally, the absorbance of the organic portion was measured at 532 nm. The concentration of MDA in the samples was extrapolated using the MDA standard curve and expressed as nmol/g tissue (42, 43).

**Glutathione content**

The basis of the method is the reaction of sulfhydryl groups with 5, 5’-dithiobis(2-nitrobenzoic acid) (DTNB) reagent. DTNB (0.04%) reacts with sulfhydryl groups, creating a colored complex with maximum absorption at 412 nm. Briefly, a 10% homogenate was first prepared with phosphate buffer, and 10% TCA was added to the samples. Then, after the samples were centrifuged, the supernatants were separated. Eventually, DTNB was added, the samples’ absorption was evaluated, and the concentration of GSH was extrapolated using the GSH standard curve and expressed as nmol/g tissue (42, 44).

Western blotting 

After removing the brain and separating the striatum, the samples were immediately stored at -80 °C for more analysis by immunoblotting technique. Before performing western blotting, all tissue proteins were extracted using lysing buffer (50 mM Tris base, 2 mM EDTA, 2 mM EGTA, 10 mM NaF, 1 mM Na₃VO₄·2H₂O, 10 mM β-Glycerophosphate, 10 mM 2-Mercaptoethanol and Sodium deoxycholate 0.2 w/v), 0.1 mM phenylmethylsulfonyl fluoride (PMSF), and 1X protease inhibitor cocktail. Then, protein absorption was measured by the Bradford method at a wavelength of 595 nm, and the total protein concentration was calculated using the calibration curve. Protein separation was performed by loading equal quantities of each sample onto a 12 (for Bax, Bcl-2, Beclin, and α-synuclein proteins) and 15% (for caspase-3 and LC3 I/II proteins) polyacrylamide gel containing 10% SDS. Then, electro-transfer was conducted to transfer separated proteins onto a polyvinylidene fluoride (PVDF) membrane (Biorad, USA). Next, the PVDF membrane was imbedded in the Tris-buffered saline with 0.1% Tween (TBST) and overnight incubated at 4 °C. Afterward, primary antibodies, including rabbit anti-Bcl-2 (1:1000 dilution, Cell Signaling #2870), anti-Bax (1:1000 dilution, Cell Signaling #2772), anti-Caspase-3 (1:1000 dilution, Cell Signaling #9665), anti-Beclin-1 (1:1000 dilution, Cell Signaling #3495), anti Lc3 (1:1000 dilution, Cell Signaling #12741), anti α-synuclein (1:1000 dilution, Cell Signaling #4179), and internal control, Mause anti β-actin (1:1000 dilution, Cell Signaling #3700), were used to detection of targeting peptides. After overnight incubation, primary antibodies were removed, and secondary antibodies such as horseradish peroxidase-conjugated anti-mouse (1:3000 dilution, Cell Signaling #7076) and anti-rabbit (1:3000 dilution, Cell Signaling #7074) antibodies were added to the membrane and incubated at room temperature for 2 hr. Eventually, after three washes, the blot appeared using a chemiluminescent (Gel Doc Alliance 4.7, UK) detection method, and the density of the blot was used to assay the protein expression level. The final concentration of each sample protein level was calculated by comparing the blot density of each sample with the blot density of its internal references (β-actin). 

***Statistical analysis***

This study used GraphPad Prism version 9 software for data analysis. All data were obtained from at least three independent experiments, and the triplicated values of each experiment were expressed as mean ± SD. The one-way ANOVA test with Tukey as a post hoc test was used to compare more than two experimental groups. Statistically, the lowest significance level was identified with the *P*-value<0.05.

**Figure 1:**
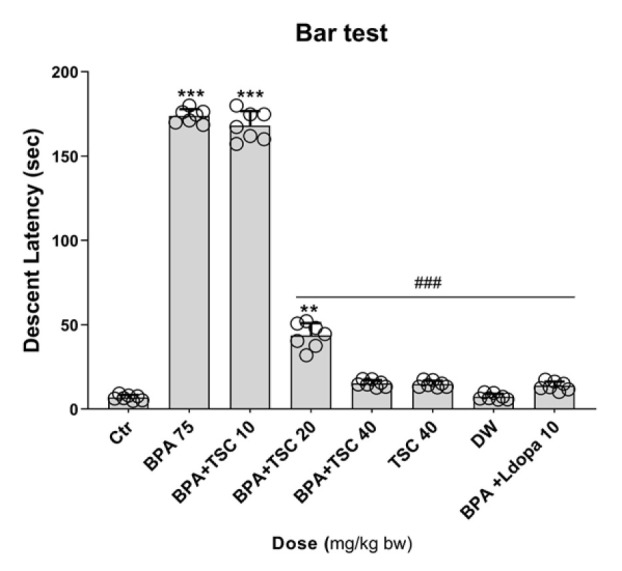
The effect of Bisphenol A (BPA) and Trans sodium crocetinate (TSC) on catalepsy in bar test in rat

Decent latency of the bar test was evaluated 24 hr after the last dose of BPA, TSC, and L-dopa, as well as olive oil and distilled water (DW) as vehicle.n=7 were used to calculate the mean. The one-way ANOVA and Tukey's post hoc test were used for data analysis, and the chart shows the values as means ± SD; **P<0.01 and ***P<.001 indicate significant differences between control and other treated groups; ###*P*<0.001 indicates significant differences between BPA and other treated groups.

**Figure 2:**
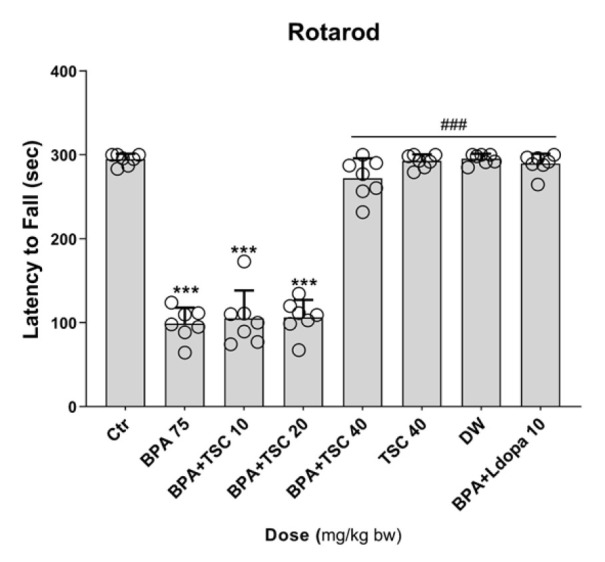
The effect of Bisphenol A (BPA) and Trans sodium crocetinate (TSC) on balance and coordination of locomotor in the rotarod test in rat

Difficulty moving and inability to balance by rotarod evaluated 24 hr after the last dose of BPA, TSC, and L-dopa, as well as olive oil and distilled water (DW) as the vehicle. n=7 were used to calculate the mean. The one-way ANOVA and Tukey's *post hoc* test were used for data analysis, and the chart shows the values as means ± SD; ***P<0.001 indicates significant differences between control and other treated groups; ###*P*<0.001 indicates significant differences between BPA and other treated groups.

**Figure 3:**
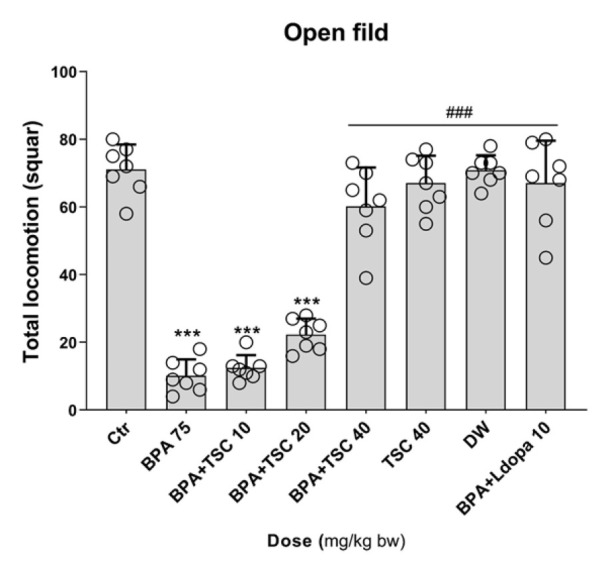
The effect of Bisphenol A (BPA) and Trans sodium crocetinate (TSC) on locomotor activities in the open field test in rat

The slowness of movements in the open field was evaluated 24 hr after the last dose of BPA, TSC, and L-dopa, as well as olive oil and distilled water (DW) as vehicle. n=7 were used to calculate the mean. The one-way ANOVA and Tukey's *post hoc *test were used for data analysis, and the chart shows the values as means ± SD; ***P<0.001 indicates significant differences between control and other treated groups; ###*P*<0.001 indicates significant differences between BPA and other treated groups.

**Figure 4:**
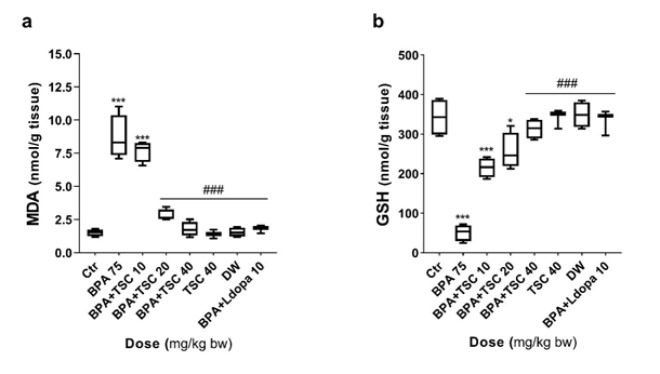
The effect of Bisphenol A (BPA) and Trans sodium crocetinate (TSC) on stress oxidative indexes in the rat brain

In the striatum region of brain tissues of rats, a) Box plot shows results for malondialdehyde (MDA) measuring using thiobarbituric acid (TBA), and b) Box plot shows results for GSH measuring using 5,5′-dithiobis-(2-nitrobenzoic acid) (DTNB). At least 3 samples were used to calculate the mean. The one-way ANOVA and Tukey's post hoc test were used for data analysis, and values are expressed as means ± SD; *P<0.05, ***P<0.001 indicate significant differences between control and other treated groups; ###*P*<0.001 indicates significant differences between BPA and other treated groups.

**Figure 5:**
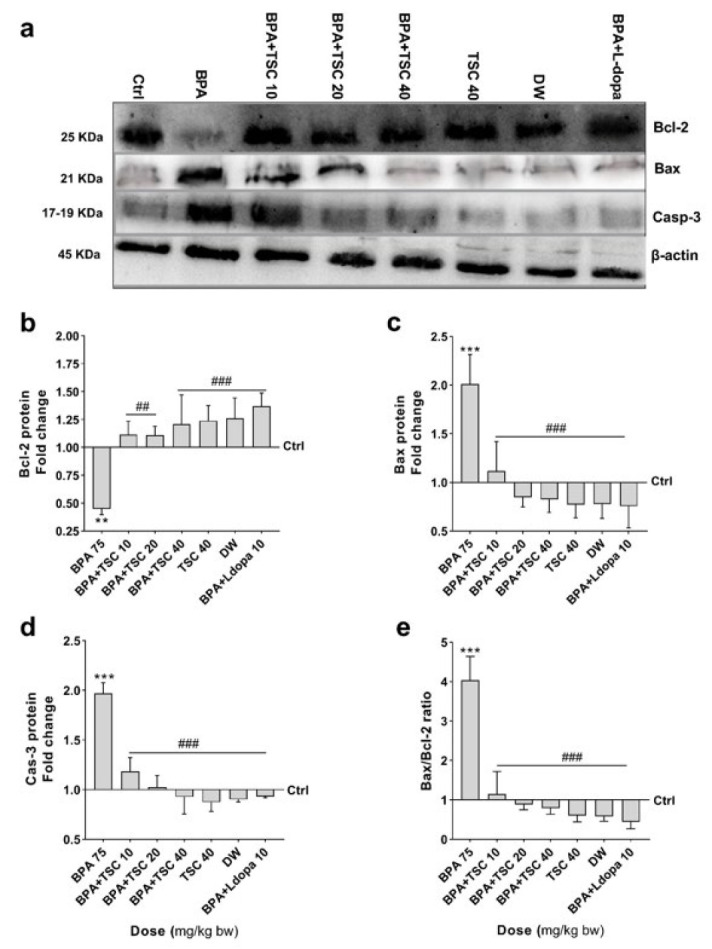
The effect of Bisphenol A (BPA) and Trans sodium crocetinate (TSC) on alpha-synuclein (α-syn) protein level in the rat brain

Western blots performed using striatum region of brain tissues of rats exposed to olive oil and distilled water (DW) (vehicle-treated controls), Bisphenol A (BPA), Trans sodium crocetinate (TSC), and L-dopa for the detection of α-syn protein; a) 18 kDa and 45 kDa bands show results for α-syn and β-actin proteins respectively, b) graph shows analysis results for α-syn protein. At least 3 samples were used to calculate the mean. The one-way ANOVA and Tukey's *post hoc* test were used for data analysis, and values are expressed as means ± SD; **P<0.01, ***P<0.001 indicate significant differences between control and other treated groups; #*P*<0.05, ##*P*<0.01 indicate significant differences between BPA and other treated groups.

**Figure 6:**
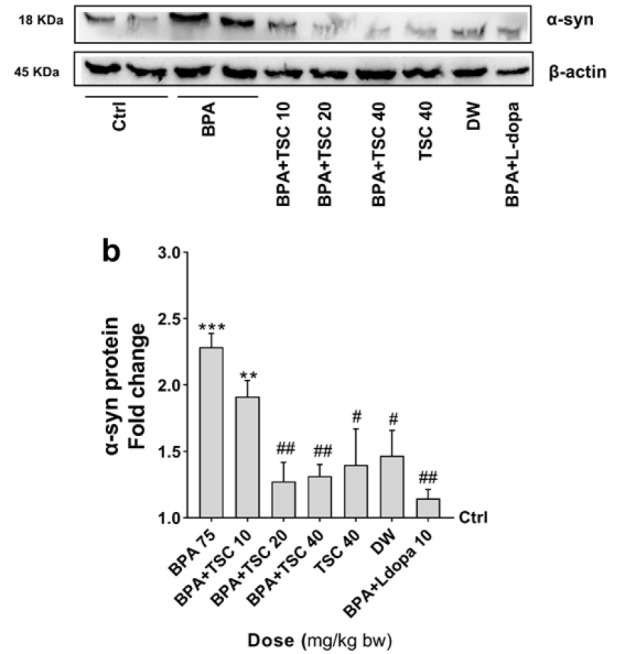
The effect of Bisphenol A (BPA) and Trans sodium crocetinate (TSC) on Bcl-2, Bax, and Caspase-3 protein levels in the rat brain

Western blots performed using striatum region of brain tissues of rats exposed to olive oil and distilled water (DW) (vehicle-treated controls), Bisphenol A (BPA), Trans sodium crocetinate (TSC), and L-dopa for the detection of apoptotic marker proteins; a) 25 kDa, 21 KDa, 17-19 KDa, and 45 KDa bands show results for Bcl-2, Bax, Caspase-3, and β-actin proteins respectively, b) graph shows analysis results for anti-apoptotic Bcl-2 protein, c) graph shows analysis results for pro-apoptotic Bax protein, d) graph shows analysis results for pro-apoptotic Caspase-3 protein, and e) graph shows analysis results for Bax/Bcl-2 proteins ratio. At least 3 samples were used to calculate the mean. The one-way ANOVA and Tukey's *post hoc* test were used for data analysis, and values are expressed as means ± SD; ***P<0.001 indicates significant differences between control and other treated groups; ##*P*<0.01, ##*P*<0.001 indicate significant differences between BPA and other treated groups.

**Figure 7:**
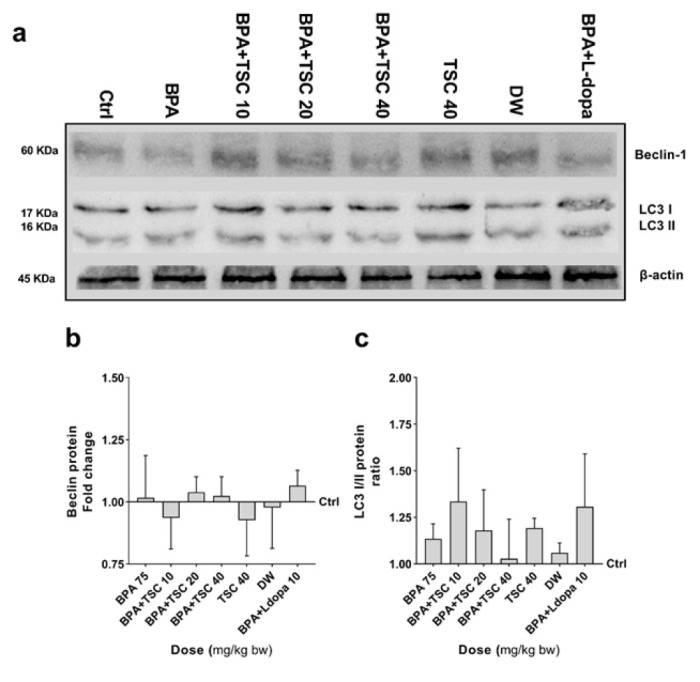
The effect of Bisphenol A (BPA) and Trans sodium crocetinate (TSC) on Beclin, LC3 I/II proteins levels in the rat brain

Western blots performed using striatum region of brain tissues of rats exposed to olive oil and distilled water (DW) (vehicle-treated controls), Bisphenol A (BPA), Trans sodium crocetinate (TSC), and L-dopa for the detection autophagy marker proteins; a) 60 kDa,16-17 KDa, and 45 KDa bands show results for Beclin, LC3 I/II, β-actin proteins respectively, b) graph shows analysis results for pro-autophagy Beclin protein, and c) graph shows analysis results for LC3 I/II protein ratio. At least 3 samples were used to calculate the mean. The one-way ANOVA and Tukey's *post hoc *test were used for data analysis, and values are expressed as means ± SD.

**Figure 8:**
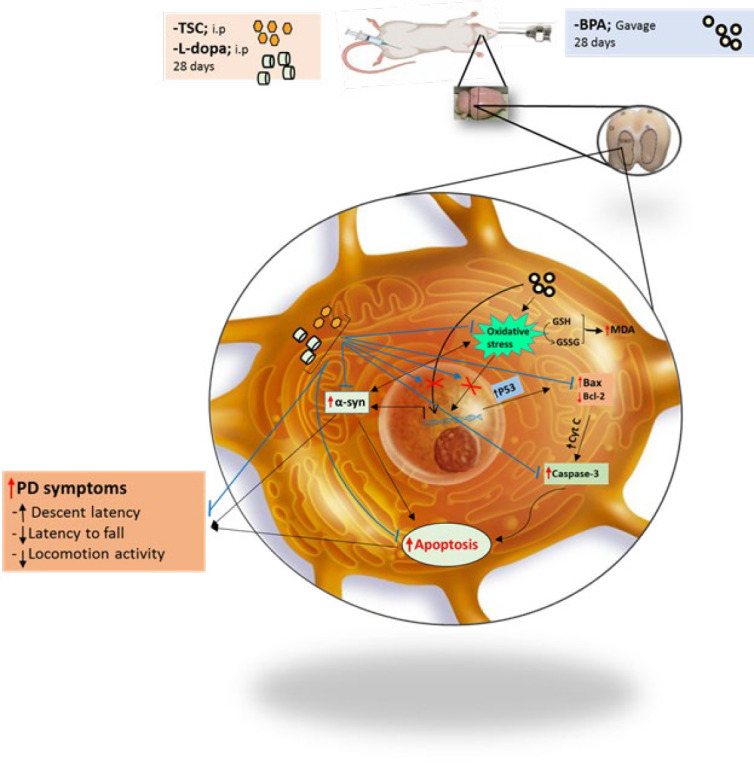
Schematic description of the mechanistic effects of Trans sodium crocetinate (TSC) and L-dopa against Bisphenol A (BPA)-induced Parkinson-like disease in the rat brain

Glutathione (GSH), malondialdehyde (MDA), α-Synuclein (α-Syn), B cell lymphoma-2 (BCL-2), Bcl-2-associated X-protein (Bax), Caspase-3.

## Results

***The effects of TSC on the BPA- induced Behavioral changes***

*TSC improved the catalepsy induced by BPA*

Our data in Figure 1 demonstrated that BPA-induced severe catalepsy was significantly decreased with TSC 40 mg/kg (*P*<0.001) compared to the BPA alone group. The catalepsy time in the rats receiving BPA alone was significantly increased compared to the control (*P*<0.001). The BPA-alone group also showed a significant increase in catalepsy time compared to the L-dopa and BPA groups (*P*<0.001). However, the group that received TSC 40 mg/kg and BPA did not exhibit a significant difference in catalepsy time compared to the L-dopa and BPA group. Moreover, the group that received TSC 40 mg/kg alone did not show a significant difference compared to the control group.

*TSC improved the BPA-induced imbalance*

Groups exposed to BPA alone showed a significant decrease in the time spent on the rotarod bar compared to the control (*P*<0.001). Our results indicated that groups receiving TSC at a dose of 40 mg/kg along with BPA exhibited a notable increase in dwell time on the rotating rod compared to the BPA-only group (*P*<0.001). There was no significant difference between the group receiving TSC 40 mg/kg with BPA and the group receiving L-dopa 10 mg/kg with BPA. In the rats that received L-dopa 10 mg/kg and BPA, the time spent on the rotarod bar significantly improved compared to those receiving BPA alone (*P*<0.001). Furthermore, the group that received TSC 40 mg/kg alone did not show a significant difference compared to the control group (Figure 2). 

*TSC ameliorated the locomotor activities of rats exposed to BPA*

Rats in groups that received BPA alone showed a statistically significant decrease in traveled distance compared to the control group (*P*<0.001), as measured by the number of squares traversed. Our analyses revealed that rats receiving TSC at 40 mg/kg and BPA exhibited a significant increase in the number of squares traversed compared to the group exposed to BPA alone. The traveled distance in the group exposed to L-dopa at 10 mg/kg and BPA significantly improved compared to the BPA-only group (*P*<0.001). The difference in distances traversed by the group receiving TSC at 40 mg/kg alone compared to control rats was not statistically significant. Furthermore, our analyses indicated that in rats receiving TSC at 40 mg/kg and BPA, there was no significant difference in the number of squares traversed compared to the L-dopa at 10 mg/kg and BPA group (Figure 3).

***Oxidative stress indices***

TSC preserved the GSH level in the brain of rats exposed to BPA

Examination of thiol groups in the striatum of rats revealed a statistically significant decrease in glutathione levels in the groups receiving BPA alone compared to the control (*P*<0.001). The changes in glutathione levels in the group receiving TSC 40 mg/kg alone were not significantly different from those in the control group. TSC 40 mg/kg alongside BPA significantly preserved glutathione resources compared to the group receiving BPA alone (*P*<0.001). The glutathione levels were also significantly preserved in the rats that received L-dopa at 10 mg/kg along with BPA alone group (*P*<0.001). However, the group that received TSC 40 mg/kg and BPA did not show a significant difference compared to the L-dopa at 10 mg/kg and BPA group (Figure 4a).

*TSC attenuated BPA-induced lipid peroxidation levels*

The assessment of MDA levels using the TBA reaction (Figure 4b) revealed a significant increase in MDA levels in the group exposed to BPA alone compared to the control (*P*<0.001). Treatment with TSC (20 and 40 mg/kg) or L-dopa (10 mg/kg) alongside the BPA group decreased MDA levels compared to the BPA-only group (*P*<0.001). However, the group receiving TSC (40 mg/kg) alone did not show significant differences in MDA levels compared to the control group (*P*<0.05). Moreover, the groups treated with TSC (20 or 40 mg/kg) and BPA exhibited no statistically significant differences in MDA levels compared to the L-dopa (10 mg/kg) and BPA group (*P*<0.001).

***Blotting analysis***

*TSC ameliorates BPA-induced α-synuclein protein expression*

The results of the blotting analysis in Figure 5 indicated a significant increase in α-syn protein levels in rats exposed to BPA alone compared to the control (*P*<0.01). The level of α-syn protein in rats receiving TSC (20 and 40 mg/kg) or L-dopa (10 mg/kg) alongside BPA significantly decreased compared to rats receiving BPA alone (*P*<0.01). However, the level of α-syn protein in the groups receiving TSC 40 mg/kg alone did not show significant changes compared to the control group. Additionally, α-syn protein levels in the groups receiving TSC 20 or 40 mg/kg and BPA did not significantly differ from those receiving L-dopa and BPA.

*TSC ameliorates BPA-induced pro-apoptotic Bax and Caspase-3 proteins and induces anti-apoptotic Bcl-2 protein expression*

The results of blot analysis revealed that in the striatum of rats exposed to BPA alone, the levels of pro-apoptotic Bax (Figure 6a,c) and Caspase-3 (Figure 6a,d) proteins significantly increased compared to the control group (*P*<0.001). In rats receiving BPA plus TSC (10, 20, and 40 mg/kg) or L-dopa 10 mg/kg, the levels of Bax and Caspase-3 proteins significantly decreased compared to the BPA alone group (*P*<0.001). Additionally, the anti-apoptotic Bcl-2 protein expression (Figure 6a,b) in the BPA alone group significantly decreased compared to the control group. Meanwhile, Bcl-2 protein levels in the groups receiving TSC (10, 20 (*P*<0.001) and 40 (*P*<0.01) mg/kg) or L-dopa 10 mg/kg (*P*<0.001) alongside BPA were significantly lower compared to rats exposed to BPA alone. However, there was no significant difference in the levels of Bax (Figure 6a,c), Caspase-3 (Figure 6a,d), and Bcl-2 (Figure 6a,b) proteins between the group receiving TSC 40 mg/kg alone and the control group. Additionally, the changes in the levels of Bax (Figure 6a,c), Caspase-3 (Figure 6a,d), and Bcl-2 (Figure 6a,b) proteins were not significant in rats treated with BPA and TSC (10, 20, and 40 mg/kg) compared to the L-dopa 10 mg/kg and BPA group.

TSC and BPA effects on the autophagy pathway proteins Beclin and LC -3 I/II expression

The expression levels of Beclin and LC-3 I/II in the groups treated with BPA, TSC, and L-dopa did not show noticeable changes compared to the control group, and there were no significant differences among these groups (Figure 7).

## Discussion

Although many factors are involved in the occurrence of Parkinson’s disease, the main factor in the occurrence of this neurodegenerative disorder is still not known. One of the strongest hypotheses for PD occurrence is the destruction of dopaminergic neurons located in the substantial nigra region resulting in decreased levels of dopamine. The death of dopaminergic neurons occurs following the accumulation of Lewy bodies containing insoluble protein α-syn in the striatum. Our findings show that TSC can ameliorate the motor symptoms of BPA-induced Parkinson-like disease by reducing the α-syn protein level and inhibiting apoptosis (Figure 8). 

Motor evaluations show that TSC has successfully prevented Parkinsonian-like symptoms such as catalepsy and movement disorders caused by involuntary muscle contractions. These observed properties of TSC were consistent with the Ajzashokouhi *et al*. study, which showed that TSC improved acrylamide-induced behavioral impairment in rats (45). The movement symptoms associated with Parkinson’s disease include tremors, muscle stiffness, slowness of movements, and difficulty in moving and walking, mainly due to a decrease in dopamine secretion resulting from the death of dopaminergic neurons in the substantia nigra region (46). Two main pathological features characterize PD. First, there is apoptosis of dopaminergic neurons caused by oxidative stress and ROS production, leading to motor stiffness, slowness, and an inability to maintain stature. Second, protein coils are formed in masses known as Lewy bodies (LBs). The primary component of these protein coils is the α-syn protein (19, 47). There is documented evidence supporting the notion that LBs, containing misfolded and accumulated α-syn proteins, induce apoptosis of dopaminergic neurons (48, 49). 

The occurrence of cytoplasmic inclusions or LBs, along with the decrease of dopaminergic neurons, are two important pathological symptoms of Parkinson’s disease (13). Destruction of dopaminergic neurons plays an important role in various neurological and locomotion disorders (50). It seems that TSC prevents the destruction of dopaminergic neurons by reducing the level of α-syn and oxidative stress and inhibiting the induction of apoptosis. In accordance, TSC has compensated for decreased locomotor activity, imbalance, and increased muscle contractions caused by BPA and prevents Parkinson’s-like symptoms.

Following TSC treatment, protein α-syn levels decreased dramatically. α-syn, which can bind to phospholipids and play a role in lipid metabolism, is a presynaptic protein of 140 amino acids implicated in PD. α-syn is highly expressed in the central nervous system (CNS), particularly in presynaptic nerve terminals. The measurement of this protein in different brain regions is used to evaluate damage caused by Parkinson’s (51). In the present study, we showed that exposure to BPA leads to an uncontrolled increase in α-syn protein levels, and treatment with TSC reverses this elevated level to the basal state. 

Accumulation of α-syn, a key component of Lewy bodies, in multiple brain regions indicates the creation of intracellular toxicity in the brain tissue. Mutations or increased protein expression are associated with Parkinson’s disease (52, 53). Lewy bodies, eosinophilic cytoplasmic spheres, lead to the aggregations of proteins such as α-syn in various brain areas, causing damage to intracellular signals and cellular respiration. However, the precise role of Lewy bodies in neuronal death is not understood (51). It seems that the use of TSC has improved BPA-induced Parkinson-like symptoms by reducing α-syn protein levels in rats. The potential mechanism of how TSC ameliorates the increased α-syn protein levels induced by BPA will be discussed in the following.

Based on the results of the present study, TSC has reversed the disturbance of BPA-induced oxidative stress indices. As shown in Figure 4a and b, TSC dramatically diminished and elevated the increased level of lipid peroxidation and decreased sulfhydryl groups caused by BPA, respectively. BPA induces ROS production by reducing the activity of antioxidant enzymes such as superoxide dismutase, catalase, and glutathione and increasing lipid peroxidation (54-56). Increased α-syn protein levels can be another possible mechanism that contributes to BPA-induced ROS production. A mutual relationship exists between α-syn levels and oxidative damage, mitochondrial cytochrome c release, and mitochondrial dysfunction (13, 57). Therefore, one hypothesis is that α-syn aggregation is increased due to the reduction of ROS-scavenging enzyme activity by BPA. This is consistent with previous studies (58, 59). It was reported that mitochondrial cytochrome c release can also be involved in α-syn aggregation (60). Excessive oxidative stress also can play a pivotal role in the pathogenesis of α-syn by promoting the α-syn misfolding and its intracellular aggregation (60-62). Conversely, abnormal a-syn aggregation can lead to an imbalance in Ca2+ homeostasis, increase cytosolic calcium and reactive oxygen and nitrogen species production, and activate calcium-dependent proteases (51, 63). However, BPA induces oxidative stress followed by an increase in α-syn accumulation, increasing free radicals. TSC can abrogate the effects of BPA on a-syn production and aggregation.

In response to the question regarding the bilateral increase of oxidative stress and the effects of α-syn on the survival of striatum region cells, this study investigated the indices of apoptosis and autophagy. The results indicate that TSC strongly suppresses BPA-induced apoptosis through a significant decrease in the pro-apoptotic proteins Bax and caspase-3 and a substantial increase in the anti-apoptotic protein Bcl-2. BPA may induce apoptosis in the cells of the striatum, both directly through excessive ROS levels and indirectly by increasing α-syn production and aggregation (55, 56, 59, 60, 62). 

 BPA might stimulate ROS-induced apoptosis in dopaminergic neurons by increasing the aggregation of α-syn proteins (49). Excessive oxidative stress, a known initiator of apoptosis in various cells and animal models, is also implicated in the development of several neurodegenerative disorders, including Parkinson’s and Alzheimer’s disease (64). Studies have shown that any method reinforcing the antioxidant system and contributing to oxidative homeostasis balance can be considered a potential treatment approach against PD (65, 66). The antioxidant effects of the main compounds of the saffron plant, such as crocin and crocetin, have been well studied. Notably, crocin has improved Parkinson ‘s-like symptoms by reducing apoptosis and α-syn protein levels in rats (2). Studies have reported that both crocin and crocetin exert their neuroprotective effects by scavenging and reducing the production of free radicals and safeguarding energy-producing pathways (2, 67, 68). Consistent with prior research, which showed that TSC is potentially a potent antioxidant and anti-apoptotic compound (69), our study indicates that TSC, an active derivative of crocetin, may prevent the onset of BPA-mediated Parkinson-like symptoms by maintaining oxidative homeostasis, preventing apoptosis, and suppressing the production and accumulation of α-syn protein.

The investigation of autophagy markers Beclin and LC3 proteins in the present study showed that removal by the autophagy process is not the mechanism involved in destroying the striatum area cells. Almost 30 genes regulate autophagy, among which LC3 and Beclin-1 genes play a pivotal role. Beclin-1 is involved in the signaling pathways and in the initiation phase of autophagosome formation, where the interaction with PI3PK and hvp34 is necessary (70-72). LC3 consists of a soluble (cytosolic) form, LC3I, and a lipid form, LC3II, expressed as three isoforms (LC3A, LC3B, LC3C) in mammalian tissues. The LC3I interacts with phosphatidylethanolamine to form the LC3II, specifically localizing on the autophagosome (71, 72). Although crocetin has been shown to induce amyloid-beta clearance by triggering autophagy through the STK11/LKB1-mediated AMPK pathway (12), the use of TSC in the present study did not have significant effects on the autophagy marker proteins. TSC apparently regulates autophagy by increasing the BCL-2 protein level, which can interact with Beclin-1.

Bcl-2/ Beclin-1 complex can prevent the development of autophagy (73). An increased expression of caspase protein caused by BPA, contributing to the apoptosis process, is also proposed as a mechanism for BPA-mediated autophagy abrogation and induced apoptosis (74). The activation of caspases cleaved the Beclin-1 proteins and inhibited the development of autophagy. The overexpression of α-syn by BPA is probably another stronger mechanism to abolish autophagy. Several studies have shown that overexpression of α-syn inhibits autophagosome synthesis and maturation (19, 75). α-Syn inhibits autophagosome synthesis by disrupting Rab1 a, a vital regulator of the secretory pathway, secretion, and homeostasis (19). Abnormal actin stabilization by α-syn is also a mechanism documented to impair autophagosome maturation (75). TSC Probably reversed the inhibitory effect of BPA on autophagy but not significantly and prevented the induction of apoptosis by ameliorating the α-syn level in the striatum cells. 

## Conclusion

Taken together, TSC prevents motor-associated functions such as locomotor activity, balance, and coordination disorder, which are the Parkinson-like symptoms caused by BPA, by inhibiting the incidence of apoptosis and neural cell death in the striatum area of the rat’s brain via 1( Prevention of ROS overproduction) and 2( Inhibition of excessive expression of α-syn protein). Therefore, considering natural productions such as TSC as a therapeutic strategy for the treatment of Parkinson’s disease can be of interest to researchers.

## Data Availability

The data supporting this study’s findings are available upon request from the corresponding author.

## Declaration

During the preparation of this work, the author(s) used Al-assisted technologies to rephrase to reduce plagiarism and improve language and grammar. After using this tool/service, the author(s) reviewed and edited the content as needed and take(s) full responsibility for the content of the publication.
